# Failure to Down-Regulate *miR-154* Expression in Early Postnatal Mouse Lung Epithelium Suppresses Alveologenesis, with Changes in Tgf-β Signaling Similar to those Induced by Exposure to Hyperoxia

**DOI:** 10.3390/cells9040859

**Published:** 2020-04-02

**Authors:** Cho-Ming Chao, Gianni Carraro, Zvonimir A. Rako, Johannes Kolck, Jamschid Sedighi, Volker Zimmermann, Alena Moiseenko, Jochen Wilhelm, Brittany M. Young, Lei Chong, Jin Wu, Adriana Contreras, Parviz Minoo, Guillermo Barreto, David Warburton, Saverio Bellusci

**Affiliations:** 1 Key laboratory of Interventional Pulmonology of Zhejiang Province, Department of Pulmonary and Critical Care Medicine, First Affiliated Hospital of Wenzhou Medical University, Wenzhou 325027, Zhejiang, China; Cho-Ming.Chao@paediat.med.uni-giessen.de; 2 Cardio-Pulmonary Institute and Institute for Lung Health, Universities of Giessen and Marburg Lung Center, Member of the German Center for Lung Research, Justus-Liebig-University Giessen, 35392 Giessen, Germany; Zvone91@web.de (Z.A.R.); johannes.kolck@charite.de (J.K.); jamschid.sedighi@gmail.com (J.S.); volkerzimmermann92@gmail.com (V.Z.); alena.v.moiseenko@gmail.com (A.M.); jochen.wilhelm@chemie.bio.uni-giessen.de (J.W.); 3 Division of General Pediatrics and Neonatology, University Children’s Hospital Gießen, Justus-Liebig-University, 35392 Giessen, Germany; 4 Cedars-Sinai Medical Center, Lung and Regenerative Medicine Institutes, Department of Medicine, Los Angeles, CA, 90027, USA; Gianni.Carraro@csmc.edu; 5 Department of Neurology, University of California, Los Angeles, CA 90095, USA; brittanym.young@gmail.com; 6 Institute of Pediatrics, Discipline of Pediatric Respiratory Medicine, Second Affiliated Hospital of Wenzhou Medical University, Wenzhou 325027, Zhejiang, China; chongpeilei@gmail.com; 7 College of Life and Environmental Sciences, Wenzhou University, Wenzhou 325027, Zhejiang, China; cugin1519@163.com; 8 Lung Cancer Epigenetics, Member of the German Center of Lung Research (Deutsches Zentrum für Lungenforschung, DZL), Max-Planck-Institute for Heart and Lung Research, 61231 Bad Nauheim, Germany; madecontreras@gmail.com (A.C.); Guillermo.Barreto@mpi-bn.mpg.de (G.B.); 9 Department of Pediatrics, Division of Newborn Medicine, University of Southern California, Children’s Hospital Los Angeles, Los Angeles, CA 90027, USA; minoo@usc.edu; 10 Brain and Lung Epigenetics (BLUE), Glycobiology, Cell Growth and Tissue Repair Research Unit (Gly-CRRET), Université Paris-Est Créteil (UPEC), 94010 Créteil, France; 11 Developmental Biology and Regenerative Medicine Program, Saban Research Institute of Children’s Hospital Los Angeles and University of Southern California, Los Angeles, CA 90027, USA

**Keywords:** *miR-154*, AT2, hyperoxia, Caveolin1, Tgf-ß1, alveolar simplification

## Abstract

Background: Bronchopulmonary dysplasia (BPD) is a lung disease of preterm born infants, characterized by alveolar simplification. MicroRNA (*miR)* are known to be involved in many biological and pathological processes in the lung. Although a changed expression has been described for several *miR* in BPD, a causal role remains to be established. Results: Our results showed that the expression level of *miR-154* increases during lung development and decreases postnatally. Further, hyperoxia treatment maintains high levels of *miR-154* in alveolar type 2 cells (AT2). We hypothesized that the decrease in *miR-154* expression in AT2 cells is required for normal alveologenesis. To test this hypothesis, we generated a novel transgenic mouse allowing doxycycline-based *miR-154* overexpression. Maintenance of *miR-154* expression in the postnatal distal lung epithelium under normoxia conditions is sufficient to reproduce the hypoalveologenesis phenotype triggered by hyperoxia. Using a pull-down assay, we identified *Caveolin1* as a key downstream target of *miR-154*. Caveolin1 protein is downregulated in response to overexpression of *miR-154*. This is associated with increased phosphorylation of Smad3 and Tgf-ß signaling. We found that AT2 cells overexpressing *miR-154* display decreased expression of AT2 markers and increased expression of AT1 markers. Conclusion: Our results suggest that down-regulation of *miR-154* in postnatal lung may function as an important physiological switch that permits the induction of the correct alveolar developmental program, while conversely, failure to down-regulate *miR-154* suppresses alveolarization, leading to the common clinically observed phenotype of alveolar simplification.

## 1. Introduction

Alveologenesis is an essential developmental phase occurring postnatally in mice and during late gestation in humans. This phase is characterized by elastin deposition in the alveolar sacs leading to the formation of “secondary septa” at the very same place of elastin deposition. The cells which are responsible for the depositing ring shaped elastin structures that surround the mouths of developing alveoli are called “alveolar myofibroblasts”. The “secondary septa” form the spheroidal walls of mature alveoli. As the process of alveolarization progresses, it leads to a marked increase of the alveolar surface and therefore the area available for gas exchange increases. The process of alveologenesis mainly takes part in the period of P0-P14 in mice (approximately equivalent to the first 6 months after birth in humans). Afterwards, from P14 through P28, the maturation of the alveoli occurs [1]. Many different signaling ligands and receptors are involved in the process of alveolarization: absence of Platelet derived growth factor alpha (Pdgfα) leads to lack of Pdgf receptor α expressing cells, which potentially form a progenitor population for the α-smooth muscle actin-expressing alveolar myofibroblasts [2,3,4]. Sonic hedgehog (Shh) signaling is also required for proper alveolar myofibroblast differentiation [1]. In addition, in a newborn murine hypoalveologenesis model, Perl et al. showed that the re-alveolarization induced by application of retinoic acid is dependent on Fgf signaling [4]. Interestingly, the absence of both *Fgfr3* and *Fgfr4* leads to impaired alveologenesis [5] but the endogenous Fgf ligands for these receptors are still unclear. During mouse lung development, increased Fgf signaling in the mesenchyme leads to impaired alveolar myofibroblast formation, associated with decreased elastin deposition [6].

Bronchopulmonary dysplasia (BPD) is the most common chronic airway disease of prematurely born infants, whereby low gestational age and weight at birth embody important factors increasing the probability of BPD occurrence (e.g., 20% of the infants born with a gestational weight of under 1500 g and a gestational age of under 30 weeks develop BPD in the US) [7,8,9,10]. Between 10,000 and 15,000 preterm infants are affected by BPD each year in the US alone [11]. The number of BPD patients increases due to improved therapy and increased survival rate at lower gestational ages [7,9,12]. From the pathophysiological point of view, BPD prevents alveologenesis from occurring. Oxygen toxicity associated with mechanical ventilation is considered as one of the major injurious factor in the pathogenesis of BPD. BPD interferes with the process of alveolarization, leading to a phenotype of alveolar simplification, which has been quantified as a decreased number but with an increased diameter of alveoli in rats [13]. Furthermore, thicker alveolar walls remain and the development of the pulmonary vasculature is disrupted [8], collectively leading to restricted gas exchange due to reduced alveolar surface and increased distance within the alveolar wall for gas diffusion between the alveolar lumen and the capillary lumen.

As more patients with BPD survive due to optimized therapy [9,14], but nevertheless carry symptoms and have impaired lung function, there is an urgent need for a better understanding of the pathophysiological mechanisms underlying this complex disease. In addition, the development of new and better diagnostic approaches (e.g., *miRNAs* as markers in peripheral blood [8]) that can potentially distinguish between prematurely born infants at risk of developing BPD will enable therapy at an early stage or prevent unnecessary therapy. Finally, new therapeutic tools will be instrumental to attenuate the symptoms of impaired lung function after surviving BPD to improve the condition of these patients and to lower the high costs of caring for infants with this disease.

MicroRNAs (*miRs*) are small regulatory RNAs in mammals that account for approximately 1% of the genome. They are 22- to 25-nucleotide-long single-stranded RNAs processed from hairpin transcripts, that regulate gene expression post transcriptionally in eukaryotes by binding at the 3′-UTR regions of the target mRNA, thus leading to mRNA cleavage, degradation or translational repression. The maturation of hairpin transcripts give rise to two isoforms, a *3p* guide strand and *5p* sister passenger strand. In general, only one isoform remains while the complementary isoform is degraded. But in some cases both isoforms can be produced thereby allowing the silencing of specific sets of genes through base pairing to a minimal recognition sequence [15]. *miRs* are involved in almost every known molecular process [16]. Yet, only little is known about their role in late lung development nor their involvement in BPD [8]. Although a changed expression has been described for several *miRs* in BPD, a causal role in BPD remains to be established [8,13].

*miRs* in general are now known to be involved in many biological and pathological processes in the lung [17]. *miR-154-3p* and *miR-154-5p* (initially called *miR-154* and miR-154* are both part of the human “*DLK1-DIO3* genomic region”, which is located on chromosome region *14q32* (murine chromosome *12F2* region) [18,19,20,21]. Among the paternally expressed imprinted genes in this genomic region, *DLK1*, *RTL1*, and *DIO3* and the maternally expressed imprinted genes *MEG3* (*Gtl2*), *MEG8* (*Rian*), and an anti-sense *RTL1* (*asRTL1*) are found. In addition, this region contains a *miR* cluster with 54 *miRs*, thus being one of the largest *miR* containing clusters in humans [18]. The *miRs* from this cluster are only expressed from the maternally inherited chromosome [20]. Furthermore none of these *miRs* binds their target *mRNAs* with full complementarity, suggesting that they may act on their targets by translational repression rather than by post-transcriptional decay [20]. The expression of the genes on the maternal chromosome *12F* in mice is regulated by so-called “*DMRs*” (differentially methylated regions) [22,23]. Various members of this cluster play roles in human pathologies [18].

*miR-154-3p* and *miR-154-5p* are highly conserved between mice and humans, which has been demonstrated by sequence equality [24] (*hsa-miR-154-3p = mmu-miR-154-3p* = 5′-AAU CAU ACA CGG UUG ACC UAU U-3′; *hsa-miR-154-5p = mmu-miR-154-5p* = 5′- UAG GUU AUC CGU GUU GCC UUC G). Within the *DLK1-DIO3* genomic region, this sequence is located in the maternally expressed imprinted intergenic region *Mirg* [19,20], which is regulated by an intergenic germline-derived differentially methylated region [19].

Similar expression profiles of these *miR*s in embryonic and adult lung tissue were found in humans and mice, indicating evolutionary conservation of these *miRs* as well as their potential functions in lung development across the two species [19].

In this study, we demonstrate that the expression level of *miR-154*, which increases during the fetal phases of fetal lung development, normally decreases postnatally. We further demonstrate that hyperoxia treatment maintains high levels of *miR-154* in alveolar type 2 cells (AT2). We therefore hypothesized that the postnatal decrease in *miR-154* expression in AT2 cells is required for normal alveologenesis. To test this hypothesis, we generated a novel transgenic mouse allowing doxycycline-based *miR-154* overexpression and analyzed the impact of overexpressing *miR-154* in the respiratory epithelium postnatally in normoxic or hyperoxic conditions. Our results indicate that down-regulation of *miR-154* in postnatal lungs may function as an important physiological switch that permits the induction of the alveolar developmental program, while conversely, failure to down-regulate *miR-154* suppresses alveolarization, leading to the common phenotype of alveolar simplification. Our results support the idea that down-regulation of *miR-154* within AT2 cells is an important driver of alveologenesis.

## 2. Material and Methods

### 2.1. Study Approval

Animal studies: all experiments were approved and performed in accordance with the guidelines from the Federal Authorities for Animal Research of the Regierungspraesidium Giessen, Hessen, Germany (Protocol 21/2013).

### 2.2. Mice

CD1 mice were crossed to generate WT pups. *FVB.Cg-Tg*(*Scgb1a1-rtTA*)*1Jaw/J* (thereafter called *Tg(Scgb1a1-rtTA)/+*) were kindly provided by Anne Karina Perl (Jacksonlab stock number 006232). These mice have been reported to target the respiratory epithelium during embryonic and postnatal stages. They were crossed with m*Tg(tet(o)miR-154)gc*) (thereafter called *Tg(tet(o)miR-154)/+*) generated for the need of this study by pronuclear injection of the expression cassette into the blastocysts. Mice were kept on the C57BL/6J background for at least 5 generations. Both genders were used. For induction of the transgene [*Tg(Scgb1a1-rtTA)*/+; *Tg(tet(o)miR154)*/+] mice were fed with food containing doxycycline (concentration 625 mg/kg, Altromin Spezialfutter GmbH & Co. KG, Lage, Germany). *[Tg(Scgb1a1-rtTA)/+; +/+]* littermates (negative for *Tg(tet(o)miR154)*) were used as control mice. Alternatively, we also used the *Rosa26^rtTA/rtTA^* mice (for generation of these mice see [25]) to drive ubiquitously *miR-154* expression in the lung from E7.5 to E18 [*Rosa26^(rtTA/rtTA)^; Tg(tet(o)miR154)/+*].

### 2.3. Hyperoxia Injury (BPD Mouse Model)

Newborn pups were subjected to hyperoxia (HOX) (85% O_2_) injury from P0-P8 in a chamber (Proox Model 110, Biospherix). To minimize oxygen toxicity and bias, nursing dams were rotated every 24 h between normoxia (NOX) and HOX. Pups and dams received food and water ad libitum.

### 2.4. Left Lobe Perfusion, Isolation and Tissue Processing, Alveolar Morphometry (Mean Linear Intercept, Air Space, Septal-Wall Thickness)

For newborn mice at P2, P5 and P8, the left lobe was perfused through the trachea with a pressure of 20 cm H_2_O with 5 mL PBS followed by 5 mL 4% PFA. For pups at E18.5 the tracheal perfusion was done by using 10 cm H_2_O with 1 mL PBS followed by 1 mL 4% PFA. The trachea was tied off with a string, and the lung was removed and placed in 4% PFA for max. 24 h at 4 °C. Lungs were then progressively dehydrated (30%, 50%, 70%, 99.6% ethanol, each 3 h) and embedded with a Leica embedding machine (EG 1150C, Leica, Wetzlar, Germany). Paraffin blocks were kept cold and 5 μm sections were generated.

For alveolar morphometry, lungs were flushed subsequently with PBS and 4% paraformaldehyde in phosphate-buffered saline (pH 7.0) at a vascular pressure of 20 cm H_2_O. Then PBS was infused via the trachea at a pressure of 20 cm H_2_O and fixed with 4% paraformaldehyde in phosphate-buffered saline (pH 7.0) via the trachea at a pressure of 20 cm H_2_O. Investigations were performed using 5 μm sections of the paraffin-embedded left lobe of the lungs. The mean linear intercept, mean air space, and mean septal wall thickness were measured after staining with hematoxylin and eosin (HE). Total scans from the left lobe were analyzed using a Leica DM6000B microscope with an automated stage according to the procedure previously described [26,27], which was implemented into the Qwin V3 software (Leica, Wetzlar, Germany). Horizontal lines (distance 40 μm) were placed across each lung section. The number of times the lines cross alveolar walls was calculated by multiplying the length of the horizontal lines and the number of lines per section then dividing by the number of intercepts. Bronchi and vessels above 50 μm in diameter were excluded prior to the computerized measurement. The air space was determined as the non-parenchymatous non-stained area. The septal wall thickness was measured as the length of the line perpendicularly crossing a septum. From the respective measurements, mean values were calculated.

### 2.5. RNA Extraction and Quantitative Real-Time RT-qPCR

After lung function measurements were taken, the right bronchus was clamped and either cranial and accessory or caudal and medial lobes were removed, placed in TRIZOL, homogenized in GentleMACs and frozen in liquid nitrogen for RNA extraction. RNA was isolated using the miRNeasy Mini Kit (Qiagen, Hilden, Germany) according to manufacturer’s instructions. RNA was reverse-transcribed (QuantiTect Reverse Transcription Kit, Cat. No. 205313, Qiagen GmbH, Hilden, Germany). cDNA was diluted to a concentration of 5 ng/μ.. Primers were designed using Roche Applied Sciences online Assay Design Tool (Roche Diagnostics Deutschland GmbH, Mannheim, Germany).

All primers were designed to span introns and blasted using NCBI software for specificity. Sybr Green Master Mix (invitrogen, Cat. No.11733-038, Carlsbad, CA, USA) was used for RT-PCR with a Roche LightCycler 480 machine (Roche Diagnostics Deutschland GmbH, Mannheim, Germany). Samples were run in triplicates using *Hprt* as a reference gene. Mouse primers are listed in supplementary data.

### 2.6. Isolation of Primary Alveolar Type II Cells and Microarray Experiments

Isolation of AT2 cells was performed as previously described [28] with few modifications. Briefly, the whole lung was perfused with 1 mL PBS through the right ventricle to remove the intrapulmonal blood cells. Lungs were perfused with 1 mL dispase through the trachea and the trachea was tied off with a string. Lungs were digested in 2 mL dispase for 30 min at 37 °C and minced. The suspension was sequentially filtered through 70, 40, and 10 μm nylon meshes and then centrifuged at 200× *g* for 10 min. The pellet was resuspended in Dulbecco’s modified eagle medium (Invitrogen, Karlsruhe, Germany), and negative selection for endothelial cells and lymphocytes/macrophages was performed by incubation on CD31- and CD45-coated Petri dishes for 45 min at 37 °C. Negative selection for fibroblasts was performed by adherence for 45 min at 37 °C on uncoated cell-culture dishes. Cell purity was analyzed in freshly isolated AT2 cells directly after isolation by epithelial cell morphology and immunofluorescence analysis with Nile red. AT2 cells used throughout this study demonstrated 95 ± 3% purity.

RNA from AT2 cells was purified using the RNeasy Mini Kit (Qiagen, Hilden, Germany) following the kit instructions. RNA quality was assessed by capillary electrophoresis using the Bioanalyzer 2100 (Agilent Technologies, Palo Alto, CA, USA). Purified total RNA was amplified and Cy5-labeled using the LIRAK kit (Agilent) following the kit instructions. Per reaction, 200 ng of total RNA was used. The Cy-labeled RNA was hybridized overnight to 8 × 60 K 60 mer oligonucleotide spotted microarray slides (Agilent Technologies, design ID 028005). Hybridization and subsequent washing and drying of the slides were performed following the Agilent hybridization protocol. The dried slides were scanned at 2 µm/pixel resolution using the InnoScan is 900 (Innopsys, Carbonne, France). Image analysis was performed with Mapix 6.5.0 software (Innopsys, 31,390 Carbonne, France), and calculated values for all spots were saved as GenePix results files. Stored data were evaluated using the R software and the limma package from BioConductor (free accessible software, www.r-project.org) [29]. Log mean spot signals were taken for further analysis. Data was quantile-normalized before averaging. Genes were ranked for differential expression using a moderated t-statistic [30]. Pathway analyses were done using gene set tests on the ranks of the t-values. We also carried out a gene microarray between experimental [*Rosa26^rtTA)/rtTA^; Tg(tet(o)miR154)/+*] and control [*Rosa26^rtTA)/rtTA^; +/+*] lungs (exposed to Dox food from E7.5 to E18) for the selection of potential mRNA targets after the pull-down assay. The data of the microarray experiment are deposited in GEO and are available through the accession number GSE141300 (please note that this SuperSerie is composed of three SubSeries).

### 2.7. Immunofluorescence Staining

Paraffin sections were deparaffinized, blocked with 3% bovine serum albumin (BSA) and 0.4% Triton X-100 [in Tris-buffered saline (TBS)] at room temperature (RT) for 1 h and then incubated with primary antibodies against Ki67 (Thermo-Scientific; 1:200), Cdh1/Ecad (BD; 1:100), pro-SFTPC (Seven Hills; 1:100) and pro+mature SFTPB (Abcam; 1:500) at RT for 1 h or at 4 °C overnight. After incubation with primary antibodies, slides were washed three times in TBST (TBS buffer + 0.1% Tween 20) for 5 min, incubated with secondary antibodies at RT for 1 h and then washed three times in TBST before being mounted with ProLong Gold Antifade Reagent with DAPI (4,6-diamidino-2-phenylindole; Invitrogen). Fluorescent images were acquired using Leica DM5500 B fluorescence microscope connected to Leica DFC360 FX camera (Leica, Wetzlar, Germany).

### 2.8. Fluorescence activated Cell Sorting

Whole lungs were isolated in ice-cold Hank’s balanced salt solution (HBSS). Then, lobes were chopped finely using sterile razor blades, digested in a 10 mL solution of 0.5% collagenase in HBSS on a heating plate (40 °C) with stirring at 700 rpm for 60 min. Once the homogenate was dissociated, the cell suspension was successively passed through 20G, 24G, and 26G needles, then strained on 70 μm and 40 μm filters. One volume HBSS was added to dilute collagenase and cell suspensions were centrifuged at 1500 rpm for 5 min to remove the enzyme solution. Cells were then resuspended in 500 μL 10% FCS in DMEM and stained with fluorochrome-labeled anti-mouse antibodies for 20 min at 4 °C (please see supplementary data), followed by washing and flow cytometric analysis with LSR Fortessa equipped with FACSDiva™ software (BD Bioscience, San Jose, CA, USA). FACS for lung epithelial progenitor cells was performed as previously described [31].

### 2.9. Fluorescence In-Situ-Hybridization (FISH)

5 µm sections of the left lobe of the lung were deparaffinized with Xylene (Carl Roth GmbH + Co. KG, Karlsruhe, Germany) and a decreasing gradient of Ethanol. After washing the slides with DEPC-PBS the section was digested with Proteinase K (peqlab, Germany). The time of incubation and the concentration of the Proteinase K added to the Proteinase K buffer were dependent on the age of the samples (P2: 1:3000 for 4 min; P5: 1:1300 for 7 min; P8: 1:1300 for 10 min). After washing with DEPC-PBS the sections were blocked with Dual endogenous enzyme block (DAKO Envision^TM^ + Dual Link System-HRP (DAB+) kit, USA) and then washed again with DEPC-PBS. Then, the sections were incubated in 0.01% Glutaraldehyde solution (Sigma-Aldrich Chemie GmbH, Germany) diluted in 4% PFA (Carl Roth GmbH & Co. KG, Germany) for 10 min followed by another washing step of DEPC-PBS. The sections were pre-incubated with miRCURY LNA^TM^ microRNA Detection Hybridization Buffer for 5 h at 54 °C before incubation with the miRCURY LNA^TM^ Detection probe (*hsa-miR-154-3p,* probe sequence: 5′-AATAGGTCAACCGTGTATGATT-3′) diluted in Hybridization Buffer (1:625) for 37 h at 54 °C. To protect the sections from drying out during incubation, they were covered with HybriWell Incubation chambers (Bio Cat, Germany). The sections were washed with a decreasing gradient of SSC (Sodium/Sodium citrate stock solution 1054.1, Roth, Germany) at 52 °C and incubated in a blocking solution (DIG Wash and Block Buffer Set, Roche Diagnostics GmbH, Germany) containing 72% DEPC water, 18% Maleic acid Buffer 10x and 10% blocking solution 10x for 30 min at room temperature. Anti-DIG-POD (ratio 1:400; Roche, Germany) and Sheep Serum (1:250; Dianova, Germany) were added to the blocking solution from the previous step and the section incubated for 4 h at room temperature. After a last washing step of DEPC-PBS, TSA^TM^-plus Fluorescein System (Perkin Elmer, Boston, MA, USA) was applied to the section for 15 h. Coverslips were mounted on the slides with Prolong^®^ Gold antifade reagent with DAPI (ProLong^TM^ Gold antifade reagent with DAPI, P36935, Waltham, MA, USA). Slides were stored at 4 °C for further analysis.

For quantification, at least 3 histological samples and 5 areas of each sample were used. The number of *miR-154-3p* positive cells (*miR-154-3p* positive and DAPI positive) was counted and compared to the total number of DAPI positive cells for both bronchiolar and alveolar epithelium. For each sample, a mean value of the number of *miR-154-3p* positive cells was calculated (*miR-154-3p* positive/DAPI positive cells in relation to DAPI positive cells). Statistical analyses were performed as previously described.

### 2.10. Pull Down Assay with Biotinylated miR-154-3p

MLE12 cells were cultured in six-well plates and transfected in triplicate with 3′-biotinylated *miR-154* (Bio-miR-154) or 3′-biotinylated scramble (Bio-scramble; Dharmacon), at a final concentration of 30 nM using Lipofectamine RNAimax (Invitrogen) following the manufacturer’s protocol. After 48 h, the cells were pelleted at 1000 rpm for 5 min. After washing twice, cell pellets were resuspended in 0.5 mL lysis buffer [50 mM Tris-HCl, 2 mM EDTA, 0.1% NP40, 10% glycerol, 2 mM EGTA, diethylpyrocarbonate (DEPC)-treated water, 50 U RNasin (Promega) and complete mini-protease inhibitor cocktail (Roche Applied Science)], and incubated at 4 °C for 10 min. The cytoplasmic extract was isolated by centrifugation at 10,000 rpm for 10 min. Streptavidin-coated magnetic beads (Invitrogen) were blocked for 1 h at 4 °C in blocking buffer (10 mM Tris-HCl pH 6.5, 1 mM EDTA, 1 mg/mL yeast tRNA and 1 mg/mL BSA) and washed twice with 1 mL washing buffer (10 mM Tris-HCl pH 6.5, 1 mM EDTA 0.5 M NaCl). Beads were resuspended in 0.5 mL washing buffer. Cytoplasmic extract was then added to the beads and incubated for 1 h at 4 °C with slow rotation. The beads were then washed five times with 1 mL washing buffer. RNA bound to the beads (pull-down RNA) or from 10% of the extract (input RNA), was isolated using Trizol reagent LS (Invitrogen). The level of mRNA in the Bio-*miR-154* or Bio-scramble control pull-down was quantified by qPCR. mRNA levels were normalized to a housekeeping gene (*Gapdh*, *H4*). The enrichment ratio of the control-normalized pull-down RNA to the control-normalized input levels was then calculated. The data for each cell line are representative of three independent experiments. The isolated RNA was processed for gene array analysis as described above. The data of the gene array experiment are deposited in GEO and are available through the accession number GSE141300.

### 2.11. Statistical Analyses

Significance was determined by two-tailed Student´s t-test using GraphPad PRISM statistical analysis software. All data are presented as mean ± SEM. Values of *p* < 0.05 were considered significant.

## 3. Results

### 3.1. Normal Postnatal Decrease of miR-154-3p Expression Is Prevented by Hyperoxia Treatment

**Figure 1:**
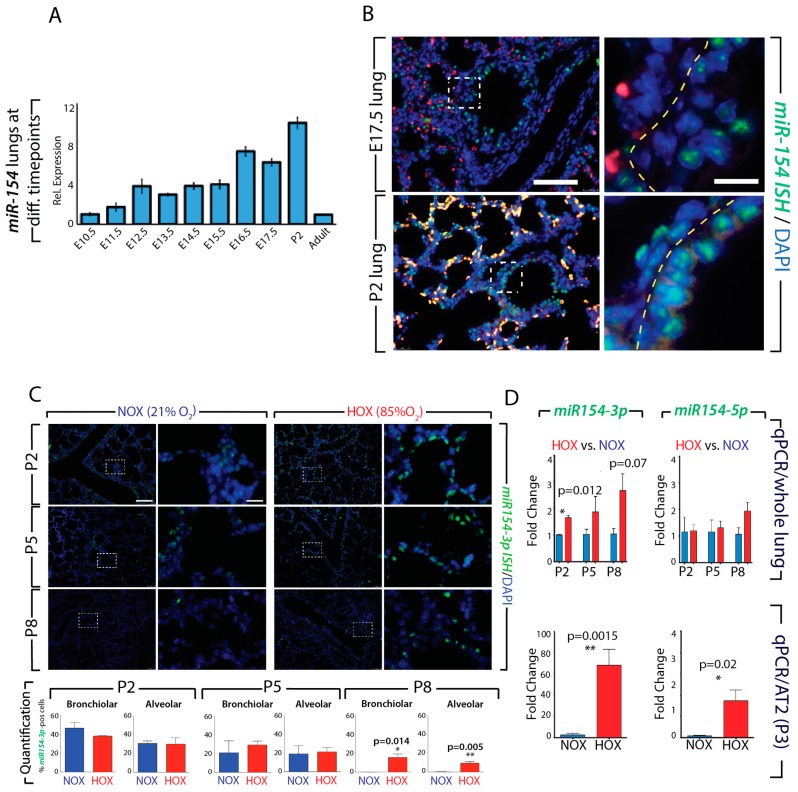
Expression of *miR-154-3p* and *miR-154-5p* during development and after hyperoxic lung injury: **A**. Expression of *miR-154-3p* in the lung from E10.5- adult age. **B**. FISH of *miR-154-3p* expression at E17.5 and P2. **C**. *miR-154-3p* expression in hyperoxic lung injury at P2, P5 and P8 by FISH and Quantification of *miR-154-3p* positive cells in bronchiolar and alveolar epithelium. **D**. *miR-154-3p* and *miR-154-5p* expression in whole lung and isolated AT2 cells by RT-qPCR. Please note: for each stage the expression of *miR-154-3p* and *-5p* in the hyperoxia have been normalized to the expression in normoxia. P2: NOX n = 2, HOX n = 3; P5: NOX n = 3, HOX n = 4; P8: NOX n = 3, HOX n = 3; P3: NOX n = 6, HOX n = 6. Scale bar in B: low magnification: 120 μm; high magnification: 30 μm. Scale bar C: low magnification: 180 μm; high magnification: 45 μm. * *p* ≤ 0.05, ** *p* ≤ 0.01, *** *p* ≤ 0.001, **** *p* ≤ 0.0001.

We have examined by qPCR, the expression of *miR-154-3p* in the embryonic lung at different developmental stages (between E10.5 and adult). Figure 1A indicates that the expression of *miR-154-3p* steadily increases during development (from E10.5 to P2) but is decreased in adult mice. Fluorescence in situ hybridization on sections show that expression *of miR-154-3p* at E17.5 and P2, is located both in the bronchiolar epithelium and in the parenchyma (Figure 1B). Exposure of neonate mice to either hyperoxia (HOX) or normoxia (NOX) for 8 days indicate that under NOX conditions, the expression of *miR-154-3p* progressively disappears from both the alveolar and bronchiolar compartment at P8. By contrast, the expression of *miR-154-3p* is maintained in these two compartments in HOX treated lungs (Figure 1C). Quantification by qPCR of the expression of *miR-154* isoforms (*miR-154-3p* and *miR-154-5p*) confirmed that at P2, the *3p* isoform was significantly increased upon HOX treatment. A trend towards an increase was also observed for this isoform at P5 and P8. No difference at the level of the whole lung was detected for the *5p* isoform (Figure 1D). Finally, we isolated alveolar type 2 (AT2) cells by FACS and detected by qPCR an increase in the expression of both isoforms in HOX versus NOX (*p* = 0.0015 and 0.02 for the *3p* isoform and *5p* isoform, respectively). These data suggest that down-regulation of *miR-154* in AT2 cells could be important to facilitate alveologenesis.

### 3.2. Generation and Validation of a Mouse Transgenic Model Allowing Postnatal miR-154 Overexpression

**Figure 2:**
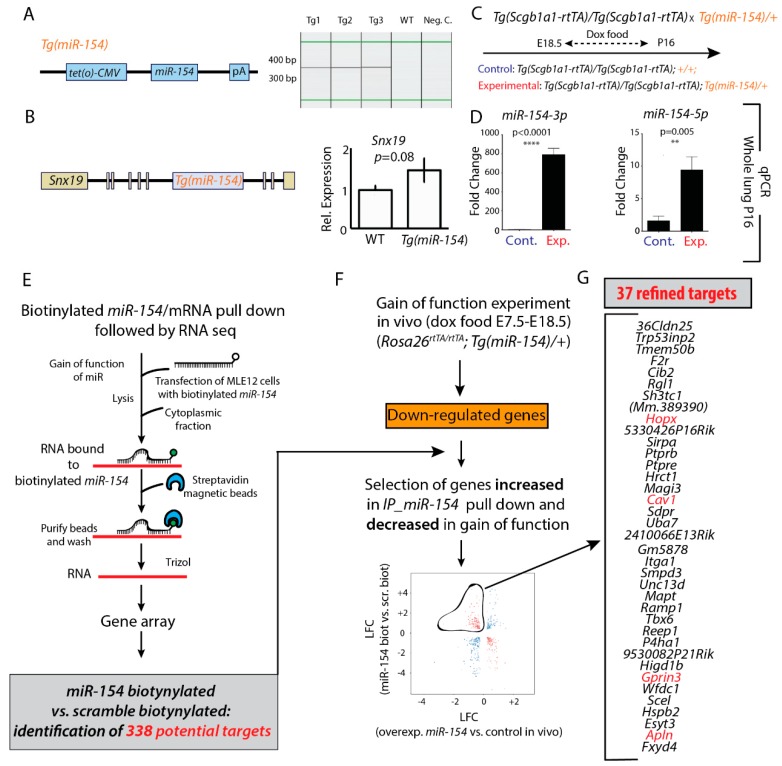
Generation/validation of transgenic mice for *miR-154* overexpression and pull-down assay. **A**. Scheme of *miR-154* transgene and genotyping results from *Tg(tet(O)miR-154)* mice. **B**. Scheme of *Snx19* gene and the Tg integration site. Expression of *Snx19* after Tg integration was analyzed by qPCR in embryonic lungs. **C**+**D**. Increased expression of *miR-154-3p* and *miR-154-5p* in double transgenic mice [*Tg((Scgb1a1-rtTA)/+*; *Tg(miR-154)/+*] versus WT [*Tg((Scgb1a1-rtTA)/+*; +/+] after Dox induction from E18 to P16. **E**–**G**. Pull down biotinylated *miR-154* versus gain of function with mimic in MLE12 cells. *[Rosa26^rtTa/rtTA^; Tg(miR-154)/+]* experimental and *[Rosa26^rtTa/rtTA^; +/+]* control embryos exposed to Dox in utero from E7.5 to E18.5 were generated and the lungs collected for genearray analysis to identify the genes downregulated upon *miR-154* overexpression. * *p* ≤ 0.05, ** *p* ≤ 0.01, *** *p* ≤ 0.001, **** *p* ≤ 0.0001.

In order to test whether down-regulation of *miR-154* is required for normal alveologenesis, we generated a mouse model allowing doxycycline-based induction of *miR-154* expression (*Tg(miR-154)*) (Figure 2A). As we used pronuclear injection as a mean to generate the transgenic animals, we next checked the site of integration of the expression cassette. We found that one copy of this cassette integrated near the *Sorting Nexin 19* (*Snx19*) gene. The integration of the *miR-154* cassette did not perturb the expression of *Snx19* (Figure 2B). To functionally validate the use of the *Tg(miR-154)* to upregulate *miR-154* expression, we crossed the *Tg(Scgb1a1-rtTA)/Tg((Scgb1a1-rtTA)* driver mice (to target the respiratory epithelium, Jacksonlab, strain number 006242) with the *Tg(miR-154)/+ mice* (Figure 2C). We exposed the pregnant females from E18.5 and the lactating mothers (with their progeny) to doxycycline food up to P16. We then collected the control [*Tg((Scgb1a1-rtTA)/+;* +/+] and experimental [*Tg((Scgb1a1-rtTA)/+;Tg(miR-154)/+*] lungs at P16. Analysis by qPCR indicates a significant upregulation of both *miR-154* isoforms in experimental versus control lungs (Figure 2D).

### 3.3. Identification of Potential miR-154 Targets Using a Biotinylated Pull-Down Assay Followed by Gene Arrays

Next, we wanted to identify the *miR-154-3p* mRNA target genes. For this purpose, we performed a pull-down experiment using a biotinylated *miR-154-3p* followed by gene array to identify these targets (Figure 2E). We used *scrambled biotinylated miR* as a control and identified 338 potential targets (deposited in GEO, accession number GSE141300). As a next step, we also identified in the *miR-154* overexpressing lungs (compared to control lungs), the downregulated gene products, which we propose may contain *miR-154* targets (Figure 2F). We overexpressed *miR-154* ubiquitously in the developing lung by exposing the pregnant females carrying both experimental [*Rosa26^rtTA)/rtTA^; Tg(tet(o)miR154)/+*] and control [*Rosa26^rtTA)/rtTA^; +/+*] embyos to Dox food from E7.5 to E18). Experimental and control E18 lungs were isolated, RNA was extracted and processed for gene arrays. The intersection between the genes identified in the pull-down assay and the genes downregulated in the lungs overexpressing ubiquitously *miR-154* allowed the identification of 37 refined targets (Figure 2G). Among them we found *Caveolin1* (*Cav1*) as well as *Homeodomain protein homeobox* (*Hopx)*, *G protein-regulated inducer of neurite outgrowth 3 (Gprin3)* and *Apelin* (*Apln*).

*Cav1* has been shown to be downregulated by Tgf-β1 via p38/MAPK, thereby inducing proliferative and anti-apoptotic properties in myofibroblasts [32]. In murine small intestine, *Fgf10* downregulates the expression of the stem cell marker *Hopx* [33]. Furthermore, and most interestingly, *Hopx* expression was shown in the bipotent alveolar epithelial cell progenitor and in AT1 cells, but not AT2 cells, suggesting *Hopx* as a marker gene for AT1 cells [34]. *Gprin3* was found to be regulated by *Fgf10* during early lung development (Bellusci and Jones, *data not published*). In humans, it was shown that APLN indirectly regulates the expression of *FGF2* and *FGFR1* in pulmonary arterial hypertension via its microRNA mediators *miR-424* and *miR-503* [35]. We propose that the regulation of some of these targets in the alveolar epithelium (mostly the AT2 cells) could be responsible for the phenotype associated with *miR-154* overexpression.

### 3.4. miR-154 Overexpression in the Lung Epithelium Postnatally under Normoxic Conditions is Sufficient to Impair Alveologenesis

**Figure 3:**
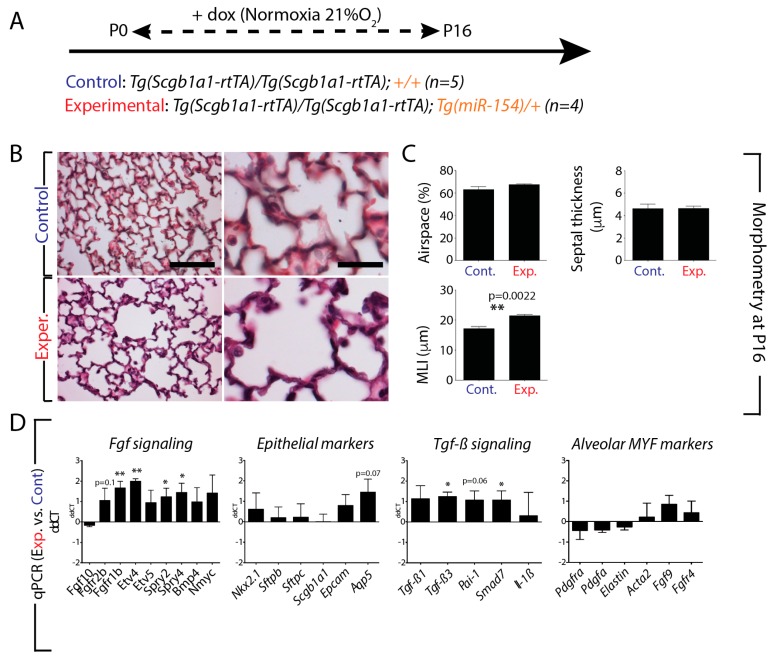
Effect of *miR-154* overexpression on lung morphology and gene expression: **A**. Model of transgenic induction of *miR-154* expression by doxycycline (Dox) administration. **B**. Experimental lungs display an increase in the size of the respiratory airway units. **C**. Alveolar Morphometry shows impaired alveolar development after *miR-154* overexpression indicated by increased MLI. **D**. RT-qPCR analysis reveals dynamically altered genetic expression profiles upon *miRNA* overexpression. *Fgf* signaling, *Tgf-β* signaling and epithelial cell markers appear to be affected in an opposite manner as under hyperoxic conditions (compare to Figure 6C), although significance is not reached concerning *Tgf-β* signaling. Interestingly, unlike hyperoxia, genes linked to alveolar myofibroblasts do not show any significant alterations upon *miRNA* Induction. Scale bar in B: low magnification: 125 μm; high magnification: 50 μm. * *p* ≤ 0.05, ** *p* ≤ 0.01, *** *p* ≤ 0.001, **** *p* ≤ 0.0001.

Our results suggested that down-regulation of *miR-154* in AT2 cells could be important to facilitate alveologenesis. In order to test this hypothesis, we generated a transgenic mouse mode where *miR-154* was induced in the alveolar epithelium upon doxycycline exposure. We used the *[Tg(Scgb1a1-rtTA)/Tg(Scgb1a1-rtTA)]* mice previously described to target the respiratory epithelium and crossed them with the *Tg(miR-154)/+* mice. We generated control *[Tg(Scgb1a1-rtTA)/Tg(Scgb1a1-rtTA); +/+]* and experimental [*Tg(Scgb1a1-rtTA)/Tg(Scgb1a1-rtTA); Tg(miR-154)/+]* neonates and exposed them from P0 to P16 to doxycycline food in NOX conditions (Figure 3A). At P16, the animals were euthanized and the lungs (n = 5 and 4 for control and experimental, respectively) were isolated for morphometry analysis as well as for gene expression. Figure 3B indicates that experimental lungs display an increase in the size of the respiratory airway units. Morphometry analysis indicates increased mean linear intercept (MLI) (*p* = 0.0022) without any significant change in airspace (*p* = 0.2111) and septal thickness (*p* = 0.9730) (Figure 3C).

Next, we used qPCR to determine potential changes in *Fgf* signaling, *Tgf-β* signaling as well as in epithelial and alveolar myofibroblasts (MYF) markers (Figure 3D). Our data suggest an increase in *Fgf* signaling upon *miR-154* overexpression in the distal epithelium Interestingly, among the significantly upregulated genes, we found *Fgfr1b* (*p* = 0.0028), *Etv4* (*p* = 0.0021) and *Sprouty4* (*p* = 0.0412), which have been described to be expressed also in the lung mesenchyme [36,37,38]. The analysis of the epithelial markers showed that *Aquaporin 5* (*Aqp5*) is increased, even though it did not reach significance (*p* = 0.0685). We also found evidence for increased *Tgf-β* signaling as indicated by upregulation of *Tgf-β3* and *Sma mother against decapentaplegic* (*Smad7)* (*p* = 0.0394). *Plasminogen activator inhibitor type 1* (*Pai-1*) as a downstream target of *Tgf-β* signaling was upregulated but did not reach statistical significance (*p* = 0.0585). In addition, we did not observe any significant change at the level of the alveolar MYF markers.

### 3.5. Identification of Genes Regulated in AT2 Cells from Experimental Versus Control Lungs and Analysis of AT2 Cell Differentiation

**Figure 4:**
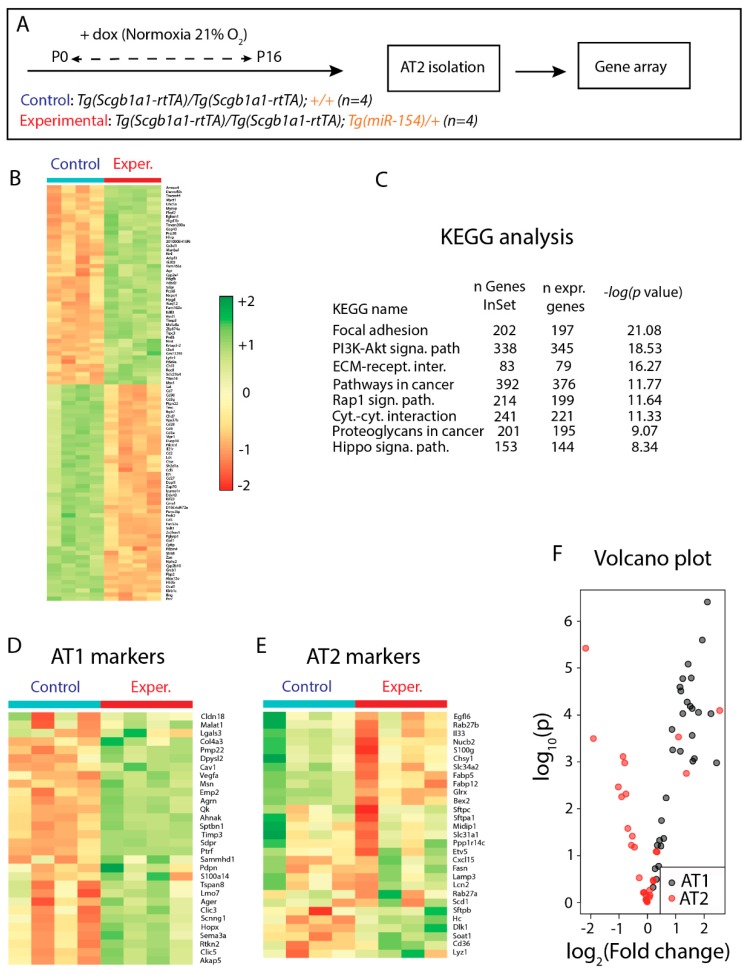
Gene Array Analysis performed on isolated AT2 samples: **A**. Model of *miR-154* induction by doxycycline administration. **B**. Differentially expressed genes upon *miRNA* overexpression. **C**. KEGG Analysis. **D**. Differential regulation of AT1 markers. AT1 markers appear to be increased in expression levels upon *miR-154* overexpression (Exper.) compared to control. **E**. Differential regulation of AT2 markers. AT2 markers appear to be inconsistently altered in expression levels upon *miR-154* overexpression (Exper.) compared to control. **F**. Volcano plot demonstrating the increased AT1 signature in isolated AT2 cells after *miRNA* induction compared to Control. For control and experimental n = 4.

At P16, at the end of the treatment with doxycycline under normoxia conditions, we isolated alveolar type 2 airway epithelial cells (AT2) by FACS from control (n = 4) and experimental (n = 4) mice and carried out gene arrays (Figure 4A). Figure 4B shows a heatmap representation of the most significantly regulated genes. KEGG analysis indicated that several pathways were significantly affected such as focal adhesion, PI3K-AKT pathway, extracellular matrix-receptor interactions and the Hippo signaling pathway (Figure 4C). Next, we monitored the expression of AT1 and AT2 markers using gene array analysis. The heatmap showed that the expression of AT1 markers, such as *Pmp22*, *Dpysl2*, *Cav1*, *Hopx* were significantly increased upon *miR-154* overexpression compared to control AT2 cells. Interestingly, the expression of AT2 markers were significantly decreased although they appeared to be inconsistently altered (Figure 4D, E). Volcano plot also indicated increased AT1 signature in AT2 cells from *miR-154* overexpressing mice compared to control AT2 cells (Figure 4F).

### 3.6. Increased Tgf-β Signaling in Experimental Versus Control Lungs in Normoxia

**Figure 5:**
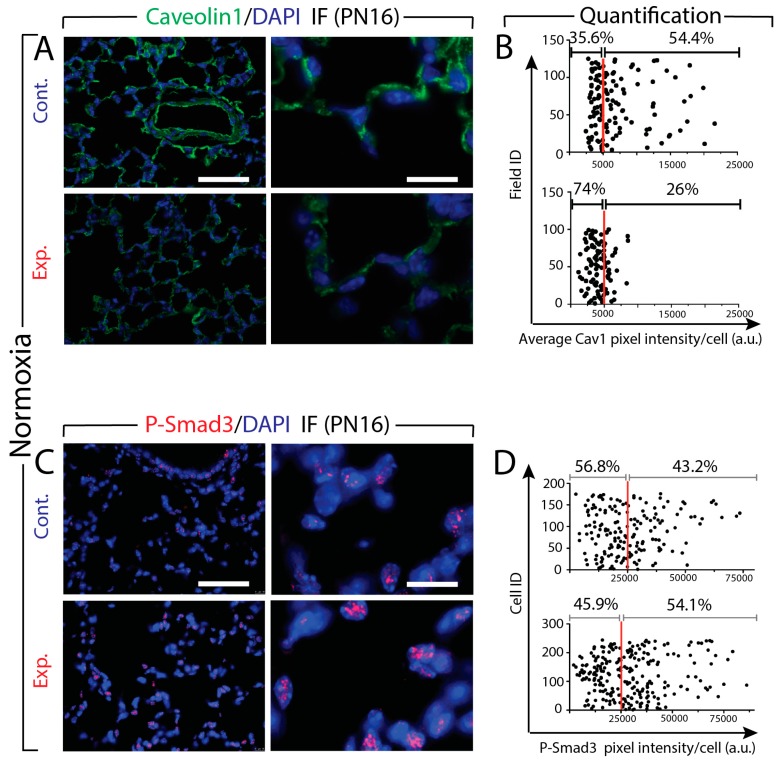
Expression of Cav1 and P-Smad3 proteins in the lung tissues from control (Cont.) and experimental (Exp.) mice at P16 under NOX condition. **A**. IF images show a decreased expression of Cav1 in the experimental lungs. **B**. Quantification of pixel intensity of Cav1 positive cells indicates a significant decrease of Cav1 expression in the experimental mice. **C**. The IF images show an increased expression of P-Smad3 in the experimental lungs. **D**. Quantification of pixel intensity of P-Smad3 positive cells supports the IF images in **C**. Scale bar **A** and **C**: low magnification: 180 μm; high magnification: 45 μm.

Two read outs of Tgf-β signaling are Caveolin1 (Cav1) and P-Smad3. Caveolin1 is a structural protein within caveolar membranes. This protein is important for caveolae formation from lipid rafts. Cav1 inhibits Tgf-β signaling by promoting Tgf-βR1 removal from the membrane and its degradation [39]. Smad3 is a downstream transducer of Tgf-β signaling as well as a transcriptional modulator. The phosphorylation of Smad3 is among the first events occurring during the activation of the Tgf-β pathway. Next, we quantified via immunofluorescence (IF) the level of expression of Cav1 and P-Smad3 in the lung tissues from control and experimental mice at P16 under NOX condition (Figure 5). Analysis of Cav1 expression indicated a significant decrease in its expression in the experimental versus control lungs (Figure 5A). Quantification of pixel intensity of Cav1-positive cells is consistent with this observation (Figure 5B). We found less cells beyond the 5000 (arbitrary units) threshold (26% versus 54%, in experimental versus control lungs, respectively). Next, we examined the expression level of P-Smad3. The IF images suggest increased expression of P-Smad3 in the experimental lungs (Figure 5C). Quantification of pixel intensity of P-Smad3 positive cells supports this observation. We found that more cells displayed increased pixel intensity beyond the 25,000 (arbitrary units) threshold (54.1% versus 43.2% in experimental versus control lungs, respectively) (Figure 5D). In addition, the dynamic range of the intensity also increased in the experimental lungs (from 25,000 to 75,000 in the control versus 25,000 to 100,000 in the experimental).

### 3.7. Upon Hyperoxia Injury, Key Signaling Pathways Were Differentially Affected between the miR-154 Overexpressing Lungs Versus Control Lungs, But the Alveolar Simplification Phenotype in Both Cases Was Morphologically Indistinguishable

**Figure 6:**
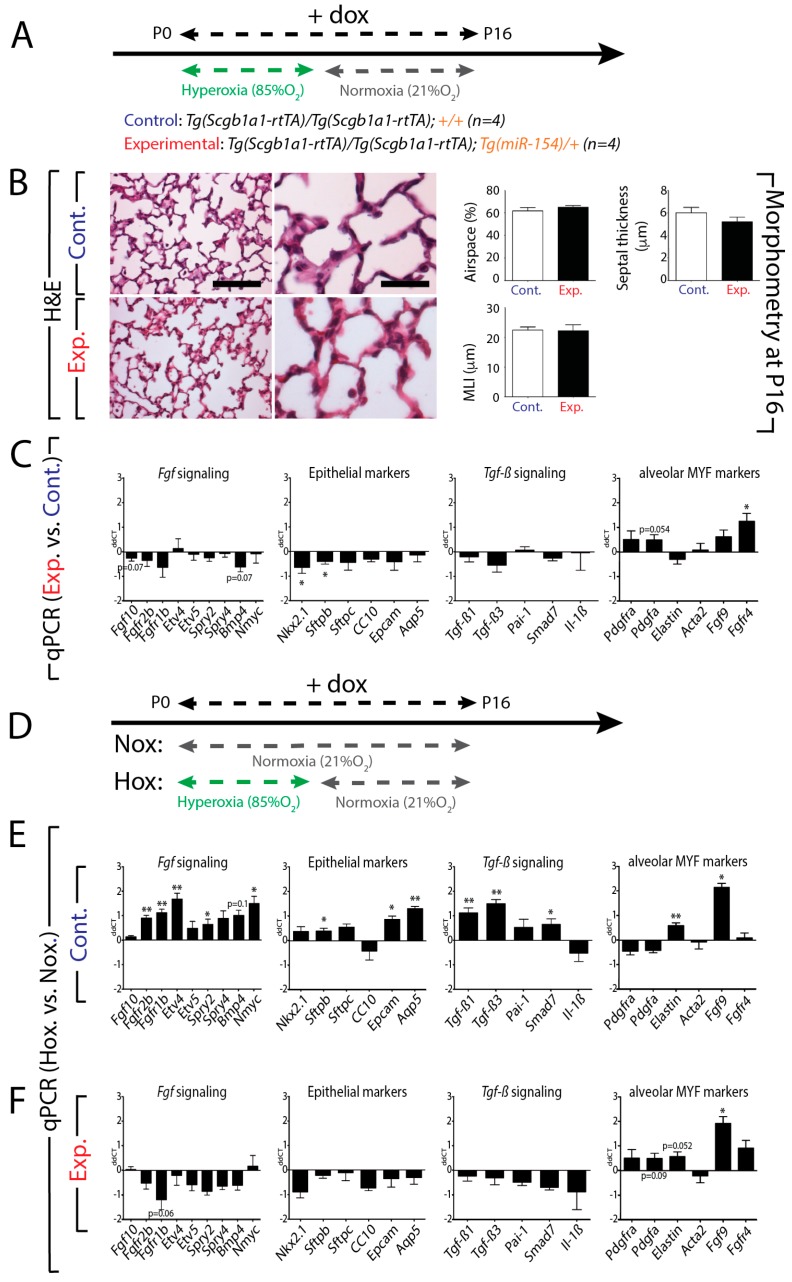
Hyperoxic lung injury on top of overexpression of *miR-154* (double injury) compared to *miRNA* overexpression only (single injury): **A**. Model of hyperoxic treatment and doxycycline (Dox) administration in order to activate *miR-154* overexpression. **B**. Alveolar Morphometry reveals no further effect of double injury (hyperoxia and *miRNA* overexpression) on alveolar parameters compared to single injury (*miRNA* overexpression only). **C**. RT-qPCR data show no obvious effect of additional hyperoxic injury on top of *miR-154* overexpression for *Fgf* signaling, *Tgf-β* signaling and epithelial cell markers. Merely genes connected to alveolar myofibroblast formation and function seem to be affected by additional hyperoxic exposure. (HOX and NOX each n = 4). **D**. Model of either normoxia or hyperoxia treatment in control and experimental mice. **E**. RT-qPCR analysis in the control mice reveals a global increase in *Fgf* signaling as well as the associated alveolar epithelial markers (*Sftpb*, *Epcam* and *Aqp5*), *Tgf-β* signaling was also increased (*Tg-fβ1*, *Tgf-β3*, *Smad7*). **F**. The data from experimental mice suggest no significant changes for *Fgf* signaling, the epithelial markers and *Tgf-β* signaling. Scale bar in B: low magnification: 125 μm; high magnification: 50 μm. * *p* ≤ 0.05, ** *p* ≤ 0.01, *** *p* ≤ 0.001, **** *p* ≤ 0.0001.

To explore the role of hyperoxic lung injury in combination with overexpression of *miR-154* on the process of alveologenesis, we exposed control and experimental mice to HOX injury for the first 8 days and then put our mice back on NOX conditions until P16 (Figure 6A). H&E and morphometric analysis indicate no significant difference between control and experimental lungs (Figure 6B). qPCR analysis using RNA extracted from the whole lung indicated a trend towards a decrease in *Fgf* signaling associated with a decrease in markers of the alveolar epithelium such as *Nkx2.1* and *Sftpb* (*Nkx2.1 p* = 0.0363; *Sftpb p* = 0.0461). For the alveolar markers, we also noticed an increase in *Fgfr4* (*p* = 0.0102) and a trend towards an increase for *Pdgfra* (*p* = 0.0535) (Figure 6C). Next, we compared the differences between HOX and NOX in control and experimental lungs (Figure 6D). Figure 6E shows the results for the control mice. We observed a global increase in *Fgf* signaling (*Fgfr2b p* = 0.0015; *Fgfr1b p* = 0.0022; *Etv4 p* = 0.0048; *Nmyc p* = 0.0188; *Spry2 p* = 0.0478) as well as the associated alveolar epithelial markers *Sftpb* (*p* = 0.0220), *Epcam* (*p* = 0.0461) and *Aqp5* (*p* = 0.0060). *Tgf-β* signaling was also increased (*Tgf-β3 p* = 0.0018; *Tgf-β1 p* = 0.0073; *Smad7 p* = 0.0439). *Elastin* and *Fgf9*, two markers of the alveolar myofibroblasts were also increased (*Elastin p* = 0.0063; *Fgf9 p* = 0.0114). Morphometric analysis indicates increased MLI as expected (data not shown). Figure 6F shows the results for experimental mice. Our results indicated that the effect of HOX normally observed in control lungs was cancelled by *miR-154* overexpression. No significant changes were observed for *Fgf* signaling, the epithelial markers and *Tgf-β* signaling. The only exception was in the alveolar markers *Elastin* and *Fgf9*, which appeared to be upregulated upon HOX exposure in the mutant lungs (*Fgf9 p* = 0.0101; *Shh p* = 0.0195; *Elastin p* = 0.0519). These results suggest that the expression of these markers is independent of *miR-154* upregulation (Figure 6F). In line with the gene expression, the morphometric analysis indicated no differences in airspace, septal wall thickness and MLI between the HOX and NOX in experimental mice (data not shown).

### 3.8. Increased Tgf-β Signaling in miR-154 Experimental Versus Control Lungs in Hyperoxia

**Figure 7:**
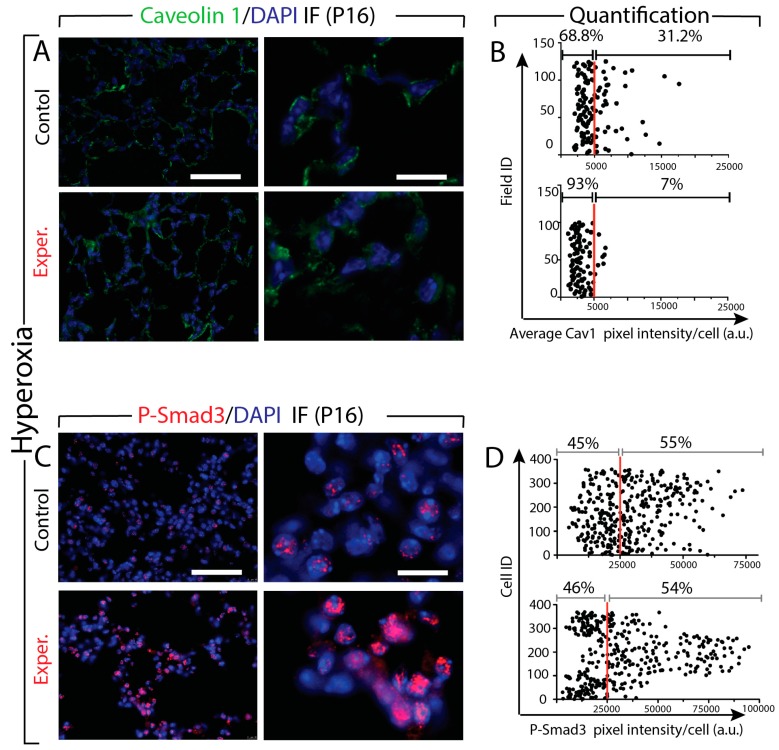
Effect of hyperoxia on the expression of Cav1 and P-Smad3 protein in the lung tissues from control and experimental mice. **A**. IF images show decrease of Caveolin1 expression. **B**. Quantification of pixel intensity of Cav1 positive cells indicates a significant decrease of Cav1 expression in the experimental mice. **C**. The IF images show an increased expression of P-Smad3 in the experimental lungs. **D**. The quantification of pixel intensity of P-Smad3 positive cells show the same percentile of cells between experimental versus control lungs. Scale bar in A and C: low magnification: 45 μm; high magnification: 180 μm.

Next, we quantified via immunofluorescence (IF) the level of expression of Cav1 and p-Smad3 in the lung tissues from control and experimental mice at P16 under HOX condition (Figure 7). The analysis of Cav1 expression indicated a significant decrease in its expression in the experimental versus control lungs (Figure 7A). Quantification of pixel intensity of Cav1 positive cells is consistent with this observation. We found less cells beyond the 5000 (arbitrary units) threshold (7% versus 31%, in experimental versus control lungs, respectively) (Figure 7B). The IF images also suggest increased expression of P-Smad3 in the experimental lungs (Figure 7C). However, the quantification of pixel intensity of P-Smad3 positive cells showed that the same percentile of cells (54% versus 55% in experimental versus control lungs, respectively) beyond the previously established 25,000 (arbitrary units) threshold (Figure 7D). Interestingly, as for the comparison of the effect of NOX (Figure 5), the dynamic range of the intensity also increased in the experimental lungs (from 25,000 to 75,000 in the control versus 25,000 to 100,000 in the experimental). Altogether, these data suggest that there is more Tgf-β signaling occurring in the experimental versus control lung but the percentile of cells showing signs of Tgf-β signaling in experimental and control lungs is the similar.

## 4. Discussion

Our data demonstrate that endogenous *miR-154* expression is normally downregulated in the distal lung epithelium after birth. However, persistent expression of *miR-154* was observed in AT2 cells isolated from postnatal lungs exposed to HOX versus NOX suggesting that downregulation of *miR-154* in AT2 could be important to facilitate the induction of alveologenesis. To test this hypothesis, we generated a novel *miR-154* gain of function transgenic mouse to overexpress *miR-154* in the airway epithelium. *miR-154* overexpression in the alveolar epithelium using the *Tg*(*Scgb1a1-rtTA*) driver line under normoxic conditions is sufficient to prevent alveolarization and triggers alveolar simplification as reflected by increased MLI. Using a pull down assay with biotinylated *miR-154*, we identified *Cav1* as a primary functional target for *miR-154*. We further demonstrated that Cav1 protein expression is decreased in *miR-154* experimental versus control lungs. This is associated with increased Tgf-β1 signaling as shown by the upregulation of P-Smad3 levels. Increased Tgf-β1 signaling in the lung at birth is associated with a BPD-like phenotype of alveolar simplification. Our conclusion that *miR-154* overexpression leads to enhanced Tgf-β signaling in the lung which could be causative for the BPD-like alveolar simplification phenotype observed in these lungs is supported by the literature. We were the first to report that gain of Tgf-β1 expression in the neonatal lung led to a BPD like phenotype [40]. This was subsequently confirmed in neonatal mice [41]. In addition, the Tgf-β signaling pathway has been shown to be upregulated in the lung of neonatal mice exposed to hyperoxia [42]. In summary, an intricate balance of Tgf-β signaling during prenatal and early postnatal mouse lung development seems to be essential for proper lung development and alveologenesis.

In addition, we report that the transcriptomic changes occurring following hyperoxia versus normoxia exposure in the control lungs (especially for Fgf and Tgf-β signaling as well as the epithelial markers) are suppressed in the *miR-154* overexpression lungs. We therefore propose that hyperoxia may be eliciting its effect on alveologenesis by suppressing the downregulation of *miR-154* expression in AT2 cells. Thus, *miR-154* down-regulation may be required to release a set of gene functions that are critical for the induction of alveolarization.

Interestingly, a link between Tgf-β signalling and *miR-154* was already described in the context of idiopathic pulmonary fibrosis (IPF). Milosevic and colleagues examined the involvement of *miR-154* in the fibrotic phenotype of IPF patients’ lungs [43]. *TGF-β1* stimulation via its downstream effector SMAD3 in vitro elicited the upregulation of various members of a *miRNA* cluster, which is mapped on human chromosome *14q32* and part of the imprinted *DLK1-DIO3* domain. This cluster included *miR-154*. *miR-154* was shown to induce proliferation and migration in lung fibroblasts partly via the repression of p15 (CDKN2B) protein level, a cell cycle inhibitor, and induction of the WNT/β-Catenin pathway.

The *Dlk1-Dio3* locus which contains *miR-154* was also described to be downstream of Histone deacetylase 3 (Hdac3). *Hdac3* knockout mice display a reduction of AT1 cell spreading leading to sacculation defects [44]. Hdac3 represses both *miR-17-92* as well as the miRNAs in the *Dlk1-Dio3* locus, which are targeting Tgf-β signaling. It was suggested that proper levels of Tgf-β signaling are important for AT1 cell remodeling. Interestingly, if Hdac3 controls the expression of both *miR-17-92* and *miR-154*, this will lead to the combined activation and repression of Tgf-β signaling. The final outcome in terms of Tgf-β signaling will depend on the level of expression of these miRs as well as on the expression of the downstream miR targets in the cells of interest. This situation is not uncommon as we previously described that *miR-142-3p* is capable of eliciting the inhibition of Wnt signaling via the targeting of the positive regulator *p300* and its activation via the targeting of the negative regulator Adenomatous polyposis coli (*Apc*) [45].

**Figure 8:**
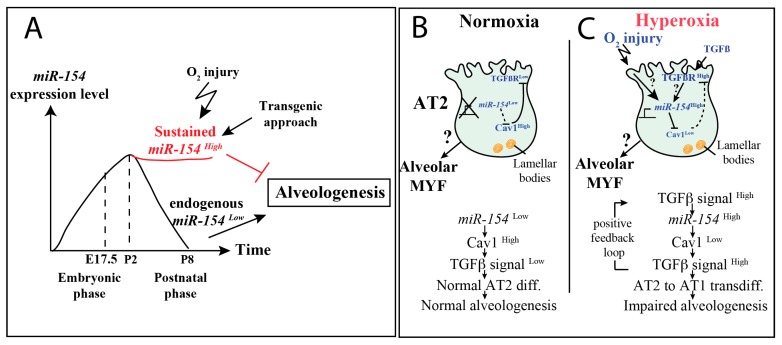
Hypothetical Model of Action describing *miR-154* function in lung development. **A.** *miR-154* expression level alterations during embryonic and postnatal phases. Postnatal *miR-154* decrease allows proper alveologenesis, whereas hyperoxia-induced sustained *miR-154* levels lead to the disturbance of alveologenesis. **B.** Under normoxic conditions postnatal *miR-154* repression occurs to enable Cav1 to inhibit Tgf-β signaling by Tgf-βr1 internalization leading to proper alveologenesis in postnatal development. **C.** Hyperoxia leads to *miR-154* induction and subsequent repression of Cav1-mediated Tgf-βr1 internalization. Tgf-β signaling is unleashed disturbing alveologenesis.

In conclusion, when considering the findings from the literature and taking into account our new results, we hypothesize that *miR-154* could also function as an important factor for embryonic development, as its expression increases during the prenatal phases towards birth and then decreases after birth (in accordance with the findings from Williams et al. [19]). However, the role of *miR-154* during fetal development is still unknown. In order to allow proper alveologenesis after birth, we also hypothesize that *miR-154* has to be decreased in expression, as both hyperoxic injury (and subsequent hyperoxia-mediated *miR-154* activation) and postnatal *miR-154* induction led to an impairment of alveolar formation also reflected in alveolar morphometric measurements (Figure 8A). Thus, the injurious effect of hyperoxic injury on alveolar formation appears to be at least partly mediated by induction of *miR-154*.

In our hypothetical model of action we hypothesize that under physiological conditions (Figure 8B) *miR-154* must be downregulated postnatally in AT2 cells, unleashing the putative target *Cav1*, which in turn leads to a downregulation of Tgf-β signaling by receptor internalization of Tgf-βr1 [39]. However, in the context of hyperoxia injury (Figure 8C), a hyperoxia-induced up-regulation of *miR-154* occurs, which inhibits Cav1-mediated Tgf-βr1 receptor internalization, thereby maintaining Tgf-β activity in AT2 cells leading to impairment of alveologenesis.

In conclusion, when *miR-154* expression in AT2 is maintained postnatally, it targets *Cav1* and thereby allows increased Tgf-β1 signaling. Increased Tgf-β1 signaling in AT2 cells in turn leads to their premature transdifferentiation towards an AT1 phenotype and this interrupts alveologenesis. Our work paves the way for the possible manipulation of the *miR-154*-Cav1-Tgf-β signaling axis to attempt circumvention of the defective alveologenesis observed in lung diseases of human prematurity such as BPD, that are characterized by alveolar simplification.

## Supplementary Materials

The following are available online at https://www.mdpi.com/2073-4409/9/4/859/s1, Figure S1: Impact of hyperoxia versus normoxia exposure of wild type mice on lung morphometry. (A) Schematic of the normoxia or hyperoxia exposure followed by normoxia and analysis at P16. (B) H&E staining of normoxia and hyperoxia lungs. (C) Morphometry measurement showing a significant increase in MLI. Scale bar in B: low magnification: 500 m; high magnification: 200 m.
