# Supplementary material for: Failure to Down-Regulate miR-154 Expression in Early Postnatal Mouse Lung Epithelium Suppresses Alveologenesis, with Changes in Tgf-β Signaling Similar to those Induced by Exposure to Hyperoxia
Source: Cells. 2020 Apr 2;9(4):859. doi: 10.3390/cells9040859 (PMC7226730; doi:10.3390/cells9040859)
Supplement: Supplementary file 1 [file cells-09-00859-s001.pdf]

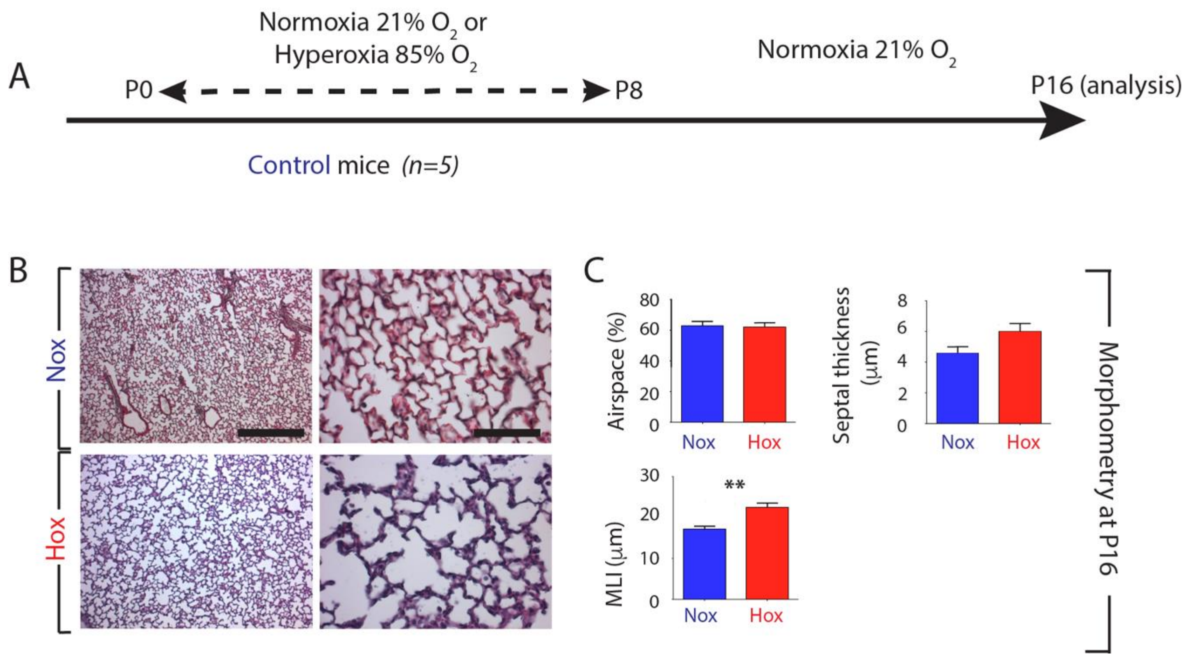

Figure for reviewer: Impact of hyperoxia versus normoxia exposure of wild type mice on lung morphometry. (**A**) Schematic of the normoxia or hyperoxia exposure followed by normoxia and analysis at P16. (**B**) H&E staining of normoxia and hyperoxia lungs. (**C**) Morphometry measurement showing a significant increase in MLI. Scale bar in B: low magnification: 500 m; high magnification: 200 m.
